# Influence of Internet Use on Happiness in China: Mediating Effects of Environmental Quality Perception and Moderating Role of Sense of Environmental Security

**DOI:** 10.3390/bs14100866

**Published:** 2024-09-25

**Authors:** Xiaorui Huang, Mingqi Fu

**Affiliations:** 1 School of International Relations, Huaqiao University, 668 Jimei Road, Xiamen 361021, China; huangxiaorui@126.com; 2 School of Public Administration, Central South University, Changsha 410012, China

**Keywords:** Internet use, happiness, environmental quality perception, sense of environmental security

## Abstract

This study aims to comprehensively examine the effects of different types of Internet use and happiness while considering the mediating role of environmental quality perception and the moderating role of a sense of environmental security. Drawing on the uses and gratifications theory, negativity bias, and social cognitive theory, the study investigates the mediating role of environmental quality perception and the moderating role of environmental security in the above relationship. Using data from 3162 respondents in the 2021 Chinese Social Survey (CSS) and Structural Equation Modeling (SEM), the study finds that Internet use for information and educational purposes significantly enhances happiness, with environmental quality perception acting as a mediator. Moreover, a moderating effect of environmental security was observed in the relationship between Internet use for educational purposes and national environmental quality perception. Specifically, the interaction between study-related Internet use and the sense of environmental security significantly and positively predicted national environmental quality perception. These findings highlight the complex interaction between Internet use, environmental factors, and happiness, offering insights into policy interventions aimed at improving Internet access and environmental awareness to enhance public mental health outcomes in China.

## 1. Introduction

The rapid proliferation of Internet use has fundamentally transformed daily life worldwide, with China at the forefront of this digital revolution. By the end of 2023, Internet penetration had reached 77.5%, encompassing over 1.092 billion users [1], making China the largest Internet user in the world. Internet usage spans various activities, such as social media, chats, and information-seeking, fulfilling both informational and social needs [2]. This unprecedented digital expansion has facilitated access to information, reshaped social interactions [3,4], and significantly impacted economic activities [5,6,7]. However, it has also introduced challenges, including Internet addiction [8,9] and exposure to negative content [10,11]. For example, many Chinese users under the age of 18 exhibit signs of excessive Internet use and dependency [12]. Despite these issues, China’s ranking on the World Happiness Index improved from 72nd in 2022 to 60th in 2024 [13], suggesting a potential link between the rapid development of information technology and national well-being. This calls for further exploration of the relationship between Internet use and happiness.

A growing body of literature has explored the relationship between Internet use and happiness in China [14,15]. However, the findings remain contradictory. Some studies highlight a positive correlation, suggesting that Internet use enhances social capital [16] and mental health. In contrast, other research links Internet use to negative psychosocial outcomes, such as loneliness and depression [17,18], which can affect individuals’ perceptions of happiness [19,20]. Given these mixed outcomes, researchers increasingly emphasize the importance of examining patterns of Internet use [21]. For instance, studies during the COVID-19 pandemic revealed the harmful effects of social media exposure on mental health [22]. Additionally, behavioral factors like environmental perception have emerged as key mediators between Internet use and mental health [23]. As individuals engage with the Internet, they are exposed to environmental information that shapes their attitudes toward environmental issues [24], which, in turn, can influence their mental health [25]. These findings underscore the complex between Internet use, environmental factors, and happiness.

Perceived environmental quality (EQP) may serve as an important mediator in the relationship between Internet use and happiness. Drawing on uses and gratifications (U&G) theory and negativity bias theory, individuals seek environmental information online to understand their surroundings, which shapes their perceptions [24]. Positive environmental information can improve psychological outcomes, while negative information may diminish them [26]. However, the mechanisms through which Internet use affects EQP and happiness are not fully understood. On one hand, researchers suggest that Internet use can negatively impact environmental satisfaction [27,28], as people often focus on environmental degradation [29]. Empirical evidence further confirms that the Internet amplifies residents’ environmental perceptions [30]. On the other hand, many studies emphasize the positive effects of perceived environmental quality on mental health [31,32] through mechanisms such as stress reduction [33,34] and cognitive restoration [35,36]. However, some findings point to a negative impact of environmental perception on well-being [37], revealing the complexity of this relationship and the need for further investigation. This study aims to explore how EQP mediates the relationship between different types of Internet use and happiness.

In addition to EQP’s mediating role, its effects may vary across individuals depending on moderating factors. One such factor is the sense of environmental security, which refers to the psychological need for safety and certainty in one’s environment [38]. This need significantly shapes people’s environmental perceptions and behaviors [32]. According to social cognitive theory, human behavior is a product of interactions among personal factors, environmental influences, and behavioral patterns [39]. As both a cognitive and emotional state, environmental security may influence how individuals interpret online environmental information, potentially moderating the impact of Internet use on EQP. Moreover, findings from Western contexts may not fully apply to China, where traditional values and rapid digital expansion influence Internet use behaviors [40]. Given the Chinese government’s efforts to address environmental issues [41], understanding how environmental security moderates the relationship between Internet use and happiness is essential. This study seeks to clarify these dynamics and deepen our understanding of how different types of Internet use affect EQP and happiness.

Although prior research has largely focused on the direct link between Internet use and happiness, there is a lack of understanding regarding the mediating and moderating mechanisms involved. To address this gap, the present study aims to examine how different types of Internet use affect happiness in China, specifically testing whether EQP mediates this relationship and whether a sense of environmental security moderates it.

This study makes several contributions to the literature. First, it is among the few to explore the mediating role of EQP between different forms of Internet use and happiness in China, a context characterized by rapid urbanization and environmental challenges [42]. Given China’s unique cultural emphasis on social status and the associated psychological stress from Internet addiction [40], this research expands the limited non-Western studies on this topic [43]. Second, while previous research mainly addresses the direct impact of Internet use on mental health, this paper investigates EQP as a key mediator, using negativity bias theory to uncover less understood mechanisms [44]. Finally, by introducing environmental security as a moderating variable within a social cognitive theory, the study provides new insights into the direct and indirect pathways through which Internet use influences happiness.

## 2. Theoretical Framework

**Figure 1:**
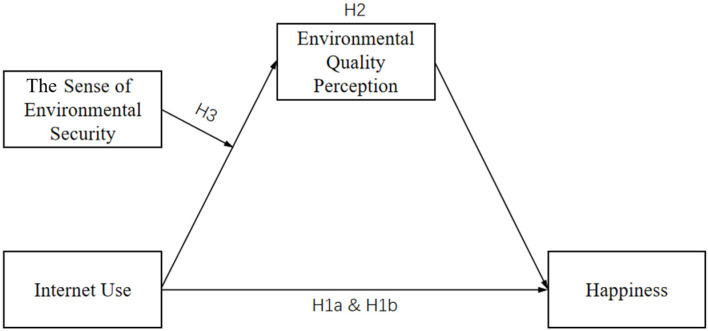
The research model.

Building on uses and gratifications theory, negativity bias theory, and social cognitive theory, we have developed a comprehensive theoretical framework to explore the relationships between Internet use, perceptions of environmental quality, and happiness among the Chinese population. Our innovative research model offers a unique perspective by focusing on the mediating role of subjective environmental quality perception and the moderating effect of the sense of environmental security. Through this framework, we explain how different patterns of Internet use can shape perceptions of environmental quality, ultimately influencing happiness (Figure 1). 

### 2.1. Uses and Gratifications (U&G) Theory

According to the U&G theory, individuals actively choose media to satisfy specific social and psychological needs, such as seeking information, entertainment, personal identity, and companionship [26]. In the case of Internet use, people engage with the medium to meet these needs, which can enhance their happiness [45]. Research has shown that Internet use has shifted individuals from being passive consumers of information to active seekers, empowering them to take greater control of their lives [46] and, in turn, boosting their well-being. Different patterns of Internet use—such as information gathering, entertainment, or social interaction—produce varied effects on happiness [47]. For example, using the Internet for information acquisition can improve efficiency, reduce information asymmetry, and increase access to diverse services and opportunities, thus creating positive welfare effects [48]. Based on this, we propose the following hypotheses:

**Hypothesis** **1a.** *Internet use is positively associated with happiness.*

**Hypothesis** **1b.** *Different types of Internet use have varying impacts on happiness.*

### 2.2. Negativity Bias Theory

Research indicates that people are more likely to focus on negative information than positive or neutral information [49,50,51]. This tendency, known as negativity bias, is an adaptive response that helps individuals recognize and avoid potential threats [51]. On the Internet, users are often drawn to negative content, especially in the context of social information exchange [52]. Numerous studies have linked negativity bias to increased levels of depression, anxiety, and a pessimistic outlook on life and the future [53]. In the case of environmental information, individuals may pay more attention to negative reports about environmental quality, leading them to perceive worsening conditions, which can decrease their happiness [54]. Thus, we hypothesize the following:

**Hypothesis** **2.** *Perceived environmental quality serves as a negative mediator in the relationship between Internet use and happiness.*

### 2.3. Social Cognitive Theory

Social cognitive theory suggests that human behavior and emotions are influenced by both personal beliefs and environmental factors [55]. A key factor in this process is the sense of environmental security, which moderates how individuals interpret and respond to environmental information encountered online. Those with a high sense of environmental security are more likely to interpret negative environmental news positively, thereby preserving a favorable perception of the environment and mitigating the adverse effects of such information on their happiness. Additionally, this theory emphasizes that individuals adopt different coping strategies when facing environmental stress [39,56]. Individuals with stronger environmental security tend to adopt proactive coping strategies, such as seeking solutions or support, which can reduce the impact of negative environmental information on happiness. Therefore, we hypothesize the following:

**Hypothesis** **3.** *The sense of environmental security moderates the relationship between Internet use, environmental quality perception, and happiness. Specifically, a higher sense of environmental security weakens the negative impact of Internet use on environmental quality perception, thereby reducing its adverse effect on happiness.*

## 3. Methods

### 3.1. Data Sources and Respondents

This study utilized data from the 2021 wave of the Chinese Social Survey (CSS), a nationwide survey initiated in 2005 by the Institute of Sociology at the Chinese Academy of Social Sciences. The CSS aims to track social changes in China by collecting longitudinal data on labor markets, family life, and public attitudes. The survey aims to provide detailed and scientific foundational data for social science research and government decision-making [57]. It provides a nationally representative sample designed to investigate the sociodemographic characteristics, employment status, living conditions, and social engagement of individuals aged 18 to 69. The 2021 survey employed a multistage, stratified, probability-proportional-to-size cluster sampling method, gathering responses from 10,136 participants across 592 villages and communities in 30 provinces and autonomous regions of China [58]. For this study, data related to Internet use, environmental factors, and happiness were selected. After removing nonstandard and incomplete responses, the final sample size was 3162. Demographic information, including age, gender, socioeconomic status (SES), and satisfaction with social welfare services, was collected to provide a comprehensive understanding of the participant pool. A total of 55.63% of the analyzed respondents were female, and 44.37% were male, reflecting the gender distribution in the sample. Regarding age, the average age was 41.407 years. Regarding the distribution of socioeconomic status (SES), 39.60% of participants were reported as middle level, 32.61% as upper-middle level, and 20.84% as upper level, indicating a predominantly middle-to-upper SES distribution in the sample.

### 3.2. Measures

**Internet use** was assessed by the frequency of engagement in various online activities, including browsing current affairs and political news, entertainment, chatting, business, and study, as identified in previous studies [59,60]. To obtain information, the CSS 2021 asked the following question: “How frequently do you use the Internet for the following activities, including browsing current affairs and political news, entertainment, chatting and making friends, business or work and study?”. Participants rated each activity on a 6-point scale ranging from 1 (“never”) to 6 (“nearly every day”). The frequency scores for browsing news (M 4.467, SD 0.48), playing (M 4.916, SD 1.591), chatting (M 4.85, SD 1.667), business (M 2.494, SD 2.128), and study (M 3.139, SD 2.045) were included.

**Happiness** was measured using a single-item, self-rating scale adapted from previous studies [61,62] and validated in Chinese [63]. Participants answered the question, “Do you feel happiness?” in CSS2021, and it is rated on a 4-point Likert-type scale, with higher scores indicating greater happiness. Single-item measures are commonly employed in happiness research [64,65] and have shown good concurrent validity [61] with the Oxford Happiness Inventory [66].

**Environmental quality perception (EQP)** was measured by respondents’ cognitive assessment of both the environmental quality in China and within their local communities, referring to previous research [29], which has been validated in Chinese. The first indicator (EQP1) asked, “What is your perception of China’s environmental quality in a global context?”. Responses ranged on a 5-point scale (1 = “low” to 5 = “high”). The second indicator (EQP2) was derived from the question, “How would you rate the environmental quality in your residential area?”, using a 10-point scale, with higher scores indicating greater satisfaction with the surrounding environment.

**The sense of environmental security** was evaluated based on respondents’ ratings of their perceived environmental security level and has been validated in Chinese [67]. The China Social Survey 2021 asked respondents the question, “How secure do you feel about the current level of environmental safety in society?”. Participants rated this on a 4-point scale ranging from 1 (“very unsafe”) to 4 (“very safe”), reflecting individuals’ feelings of insecurity in relation to their environmental surroundings.

**Covariates** included age, age square, gender, socioeconomic status (SES), and satisfaction with social welfare services, as these have been linked to happiness in previous studies [68,69,70]. The control variables included age (M = 41.407, SD = 14.12), gender (M = 0.444, SD = 0.497), SES (M = 3.668, SD = 0.894), and satisfaction with social welfare services (M = 7.265, SD = 2.162).

### 3.3. Statistical Strategies

Descriptive analyses were performed for all variables. Subsequently, we used a multivariate regression model to examine the relationship between various types of Internet use, EQP, and individuals’ happiness. This analysis controlled for various covariates, including age, age square, gender, SES, and satisfaction with social welfare services. Regressions were conducted for the total sample, with standardized coefficients and standard error reported. Additionally, to explore the mediating role of EQP and the moderating role of the sense of environmental security, we verify various moderated mediation models based on the bootstrap method to obtain point estimates of the coefficients. By taking 5000 bootstrap samples, the robust standard error and bootstrap confidence interval of parameter estimation were obtained. All analyses were conducted using Stata version 17.0.

## 4. Results

### 4.1. Descriptive Statistics

Table 1 summarizes the descriptive statistics for the 3162 respondents. The average age was 41.407 years (SD = 13.705). On average, participants rated their satisfaction with social welfare services at 7.265, while their socioeconomic status (SES) averaged 3.67, indicating a level above the median. In 2021, most respondents used the Internet multiple times per week for activities such as gaming, chatting, and news browsing. The mean score for EQP1 and EQP2 were 3.826 and 7.427, respectively. The overall happiness level was relatively high, with a mean of 3.354 (SD = 0.649). The average sense of environmental security was 3.074.

Table 2 shows significant correlations between Internet use, EQP1, EQP2, sense of environmental security, and happiness. Internet use for education had a negative association with EQP1 but was positively related to both EQP2 and happiness. Both EQP1 and EQP2 were also positively correlated with happiness. Moreover, a stronger sense of environmental security was positively linked to EQP1, EQP2, and happiness.

### 4.2. Multivariate Regression Analysis

As presented in Table 3, the results from regression models suggest that happiness is influenced by many factors, including types of Internet use, EQP, sense of environmental security, and socioeconomic characteristics of respondents. These findings imply that digital behavior, environmental perceptions, and socioeconomic status all contribute significantly to happiness.

Table 3 explores the relationship between different types of Internet use, EQP1, EQP2, sense of environmental security, and individuals’ happiness across five models (Models 1–5). Columns (1), (3), (5), (7), and (9) show the impact of various types of Internet use on happiness, incorporating EQP1 and the control variables. Meanwhile, Columns (2), (4), (6), (8), and (10) assess the effect of different types of Internet use on happiness, introducing EQP2 and the control variables.

Model 1 and Model 5 reveal that frequent Internet use, particularly for browsing news and learning, positively correlates with higher happiness, supporting Hypotheses 1a and 1b. EQP1’s impact on happiness is positive and statistically significant at the 5% level, and EQP2’s effect on happiness is also significantly positive, aligning with the previous finding [71]. Additionally, a strong sense of environmental security significantly enhances happiness, as evidenced by significant coefficients in Columns (1) (beta = 0.108, *p* < 0.001), (2) (beta = 0.101, *p* < 0.001), (9) (beta = 0.107, *p* < 0.001), and (10) (beta = 0.100, *p* < 0.001). These findings highlight the critical role environmental security plays in shaping happiness.

The estimation results for the influence of the control variables on happiness basically conform to previous findings [29]. Specifically, males and individuals with higher SES reported lower levels of happiness, while greater satisfaction with social welfare services was linked to increased happiness.

### 4.3. Testing the Mediating Effects of EQP1 and EQP2

This study posits that the different types of Internet use, such as news browsing and studying, influence happiness through the mediating roles of EQP1 and EQP2. Panel A of Table 4 shows that Internet use for studying has a significant negative effect on EQP1 (beta = −0.016, *p* < 0.05), while EQP1 significantly enhances happiness (beta = 0.052, *p* < 0.001). When both study-related internet use and EQP1 are included, Internet use for studying significantly positively impacts happiness (beta = 0.017, *p* < 0.01). The Bootstrap method was used with 5000 samples, with regression results shown in Panel C of Table 4. The bias-corrected 95% confidence interval for EQP1’s mediating effect is [−0.0020729, −0.0000925], excluding zero and confirming EQP1’s significant mediating role.

Panel B presents results for EQP2’s mediating effect. Internet use for studying significantly affects EQP2 (beta = 0.031, *p* < 0.10), and EQP2 positively influences happiness (beta = 0.031, *p* < 0.001). Even with both variables included, study-related Internet use continues to significantly impact happiness, though the coefficient is reduced (beta = 0.015, *p* < 0.01). The bias-corrected 95% confidence interval for EQP2’s mediating effect is [0.0000344, 0.002255], confirming a significant mediating role for EQP2 and supporting Hypothesis 2.

### 4.4. Testing for Moderated Mediation

**Figure 2:**
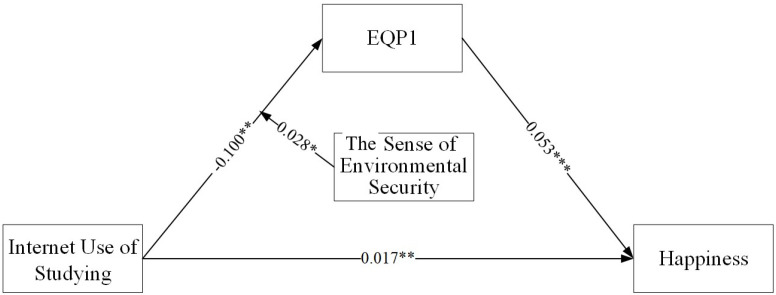
The Moderated mediated model. Note: ** *p* < 0.01, *** *p* < 0.001.

Table 5 illustrates the moderating effect of environmental security on the mediation process. The interaction between study-related Internet use and environmental security significantly predicts EQP1 (beta = 0.0280, *p* < 0.05), indicating that environmental security moderates the first half of the mediation between studying and happiness, supporting Hypothesis 3. The model fit indices indicate good fit (CFI = 0.973, TLI = 0.769, RMSEA = 0.067, SRMR = 0.011). The specific model is shown in Figure 2.

To further elucidate how the sense of environmental security moderates the influence of online education on happiness, we classified the sense of environmental security into high, medium, and low groups based on one standard deviation. We then calculated the mediating effect of the sense of environmental security between study-related Internet use and happiness, along with its 95% bootstrap confidence interval (CI) (Table 6). The mediating effect of EQP1 in the high sense of environmental security group (beta = 0.994, CI = [0.264, 1.584]) was found to be stronger than that in the low sense of environmental security group (beta = 0.740, CI = [0.213, 1.224]).

**Figure 3:**
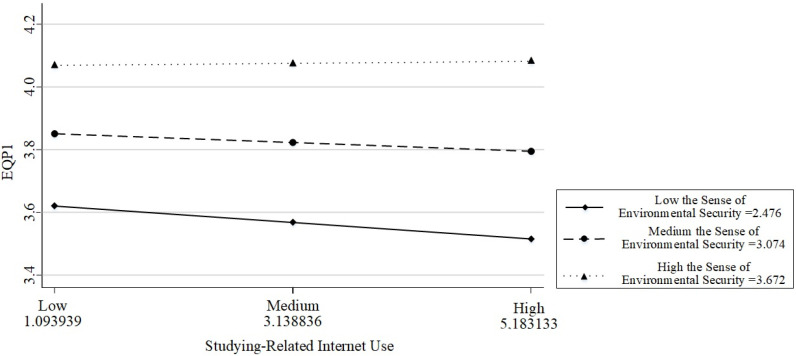
Moderating effect of the sense of environmental security on Internet use of studying and EQP1.

Based on a comprehensive analysis of the results, the moderated mediation model proposed in this study is supported. Internet use for educational purposes not only directly predicts happiness but also does so indirectly through the mediation of EQP1. Moreover, the first half of this mediation is moderated by the sense of environmental security. To better understand the interaction effect between Internet use for studying and the sense of environmental security, we categorized the sense of environmental security into high, medium, and low groups and plotted a simple effects analysis graph (see Figure 3).

The simple slope test indicates that when the sense of environmental security is low, Internet use for studying negatively affects EQP1 (beta = −0.031, *p* = 0.004); when the sense of environmental security is moderate, the impact still remains negative but weak (beta = −0.014, *p* = 0.062); and when the sense of environmental security is high, the predictive effect becomes non-significant (beta = 0.002, *p* = 0.806). These results indicate that individuals with low environmental security may experience a negative impact on their perception of environmental quality from study-related Internet use, but this effect diminishes as their sense of security increases. This highlights the complex interaction between digital behavior, environmental perceptions, and security in shaping happiness.

## 5. Discussion

The existing literature offers limited insight into the role of environmental factors in the relationship between Internet use and happiness. This study addresses this gap by examining how environmental quality perception (EQP) mediates and how the sense of environmental security moderates the effects of different types of Internet use on happiness. We apply the U&G theory, negativity bias theory, and social cognitive theory to explore these dynamics, yielding several findings worthy of discussion.

First, we found that individuals who frequently use the Internet for browsing news and educational purposes experience higher levels of happiness. This result is consistent with previous research [17] on the complex relationship between Internet use and mental health. However, this study goes further by showing the distinct effects of specific online activities, particularly news consumption and educational engagement. News browsing reduces information asymmetry and search costs, providing access to a broader range of information, affordable products, and job opportunities, all of which enhance mental well-being [72,73]. Likewise, using the Internet for educational purposes offers flexibility, enabling people to develop life skills, improve self-care, and strengthen social relationships, contributing to increased happiness [74].

Second, the study discovered that EQP mediated the link between study-related Internet use and happiness, offering new insights into how Internet use shapes perceptions of the environment and happiness. Specifically, Internet use for studying was linked to heightened awareness of local environmental quality but reduced perceptions of national environmental conditions. This suggests that study-related Internet use helps people focus on their immediate surroundings, aligning with their goals [60], while national environmental issues, often framed negatively by the media, lead to lower perceptions of broader environmental quality due to negativity bias [75].

Third, our study found that the sense of environmental security moderated the indirect relationship between study-related Internet use and happiness through national EQP, consistent with previous research on environmental risk vulnerability [76]. This influence may occur through two channels. From the emotional regulation perspective, a high sense of environmental security helps individuals effectively manage their emotions, reducing the anxiety and stress caused by Internet information and fostering positive cognition [77]. Conversely, individuals with low environmental security are more susceptible to negative information online, resulting in negative perceptions of environmental quality and decreased happiness [78]. From the self-efficacy perspective, individuals with low environmental security often have lower self-efficacy, making them more prone to frustration, which further diminishes their perception of environmental quality and happiness [79]. These findings highlight the complex interactions between digital behavior, environmental perceptions, and security feelings in shaping individuals’ happiness.

This study makes significant contributions to the current scholarly literature. First, it uses micro-level data from China to provide empirical evidence on how environmental factors, such as EQP and environmental security, influence the relationship between different types of Internet use and happiness. By doing so, we address a key gap in the literature, demonstrating how specific Internet activities impact happiness via these environmental factors. Second, this study proposes a new analytical framework by integrating U&G theory, negativity bias theory, and social cognitive theory. This framework extends the application of these theories to environmental contexts, offering a fresh perspective on how negative media content influences environmental perceptions and happiness. While traditional U&G theory focuses on how individuals actively use media to meet their needs [26], our study shows that it also applies to the processing of environmental information encountered online. Media negativity bias affects environmental quality perceptions, with potential negative impacts on happiness. Our findings broaden the scope of U&G theory, encompassing both active information-seeking behaviors and passive information processing. Third, by incorporating U&G theory, negativity bias theory, and social cognitive theory, this study validates the applicability of these theoretical frameworks to understanding how digital behaviors influence happiness. Our analysis emphasizes the role of environmental factors in shaping the psychological outcomes of Internet use, offering a more comprehensive view of the interplay between online activities and happiness.

This research offers important practical implications. First, the study demonstrates that Internet use, particularly for educational and information-seeking purposes, can positively impact happiness, supporting previous findings that link Internet use to mental health benefits [14]. Policymakers should consider enhancing access to high-quality online educational resources and creating digital platforms that provide supportive, uplifting content to promote well-being. Second, the findings show that environmental quality perception (EQP) mediates the relationship between study-related Internet use and happiness. This underscores the need to address how study-related internet activities affect both local and national environmental perceptions. Policymakers should focus on increasing public awareness and education on environmental issues through digital platforms. Developing online resources that offer accurate, constructive information about local and national environmental conditions can help balance public perceptions. Incorporating environmental education into digital literacy programs can also empower individuals to understand better and positively influence their views on residential and national environmental quality. Third, the study suggests that fostering a strong sense of environmental security can decrease the negative impact of study-related Internet use on national environmental perceptions. Government agencies should collaborate with multiple stakeholders to create educational programs that build emotional resilience [80] and self-efficacy in relation to environmental issues. By equipping individuals with skills to manage environmental risks and uncertainties, these programs can enhance their perceptions of environmental quality and, in turn, improve their overall happiness.

## 6. Conclusions

This study, based on a sample of 3162 adults from China, is among the first to explore the mediating role of environmental quality perception (EQP) and the moderating effect of environmental security in the relationship between various types of Internet use and happiness. The findings indicate that Internet use for educational purposes is positively associated with increased happiness. This relationship is partially mediated by improved perceptions of residential environmental quality and reduced perceptions of national environmental quality, with environmental security acting as a key moderating factor. These results highlight the significant influence of environmental factors on mental health outcomes, particularly in the context of rapidly evolving information and communication technologies.

However, several limitations should be acknowledged. First, as a cross-sectional study, it provides valuable insights into the mediating processes [81] but cannot establish causality. Future research should employ longitudinal data to deepen our understanding of how Internet use impacts happiness over time. Additionally, this study was limited to computer-based Internet use due to the available data, which restricts the scope of the analysis. Future research could broaden this perspective by examining the effects of various forms of Internet use, such as social media, mobile applications, and generative AI, to explore how Internet use contributes to happiness from an environmental perspective.

## Figures and Tables

**Table 1:** Descriptive statistics of variables (N = 3162).

Variables	Mean or %	Std. Dev.	Min.	Max.
Happiness	3.354	0.649	1	4
Internet use				
(a) Browsing news	4.467	1.773	1	6
(b) Playing	4.916	1.591	1	6
(c) Chatting	4.851	1.667	1	6
(d) Business	2.494	2.128	1	6
(e) Study	3.139	2.045	1	6
EQP				
EQP1	3.826	0.888	1	5
EQP2	7.427	1.976	1	10
Sense of environmental security	3.074	0.598	1	4
Age	41.407	13.705	18	70
Age square	1902.311	1164.332	324	4900
Gender	0.444	0.497	0	1
SES	3.668	0.894	1	5
Satisfaction with social welfare services	7.265	2.162	1	10

Note: All of the variables appear in the final model.

**Table 2:** Correlation matrix for variables in the model.

Variable	1	2	3	4	5	6	7	8	9	10	11	12	13	14
1. Age	—													
2. Age square	0.987 ***	—												
3. Gender	0.062 ***	0.069 ***	—											
4. SES	−0.027	−0.033 *	0.046 ***	—										
5. Satisfaction with social welfare services	−0.039 **	−0.027	0.007	−0.185 ***	—									
6. Browsing news	−0.014	−0.011	0.219 ***	−0.108 ***	0.096 ***	—								
7. Playing	−0.249 ***	−0.240 ***	−0.071 ***	−0.045 **	0.016	0.124 ***	—							
8. Chatting	−0.306 ***	−0.299 ***	−0.088 ***	−0.054 ***	0.027	0.095 ***	0.234 ***	—						
9. Business	−0.262 ***	−0.279 ***	0.079 ***	−0.073 ***	0.026	0.181 ***	0.101 ***	0.165 ***	—					
10. Study	−0.357 ***	−0.347 ***	−0.023	−0.106 ***	0.093 ***	0.319 ***	0.121 ***	0.198 ***	0.379 ***	—				
11. EQP1	0.083 ***	0.092 ***	0.037 **	−0.042 **	0.291 ***	0.021	−0.064 ***	−0.006	−0.046 ***	−0.038 **	—			
12. EQP2	0.022	0.020	−0.005	−0.147 ***	0.405 ***	0.058 ***	−0.022	0.010	−0.022	0.058 ***	0.305 ***	—		
13. Sense of environmental security	0.056 ***	0.059 ***	0.019	−0.086 ***	0.255 ***	−0.016	−0.055 ***	−0.006	−0.041 **	−0.016	0.339 ***	0.387 ***	—	
14. Happiness	0.063 ***	0.070 ***	−0.049 ***	−0.227 ***	0.283 ***	0.064 ***	0.014	0.022	−0.004	0.061 ***	0.151 ***	0.207 ***	0.185 ***	—

Note: * *p* < 0.05, ** *p* < 0.01, *** *p* < 0.001.

**Table 3:** Multivariate linear regression models predicting happiness.

	Models 1 Happiness		Models 2 Happiness		Models 3 Happiness		Models 4 Happiness		Model 5 Happiness	
	(1)	(2)	(3)	(4)	(5)	(6)	(7)	(8)	(9)	(10)
Internet use										
Browsing news	0.015 *	0.014 *								
	(0.006)	(0.006)								
Playing			0.009	0.009						
			(0.007)	(0.007)						
Chatting					0.009	0.009				
					(0.007)	(0.007)				
Business							0.002	0.003		
							(0.005)	(0.005)		
Study									0.017 **	0.016 **
									(0.006)	(0.006)
EQP										
EQP1	0.031 *		0.032 *		0.031 *		0.032 *		0.033 *	
	(0.013)		(0.013)		(0.013)		(0.013)		(0.013)	
EQP2		0.021 **		0.021 ***		0.021 ***		0.021 ***		0.020 **
		(0.006)		(0.006)		(0.006)		(0.006)		(0.006)
Sense of environmental security	0.108 ***	0.101 ***	0.107 ***	0.099 ***	0.106 ***	0.098 ***	0.106 ***	0.098 ***	0.107 ***	0.100 ***
	(0.019)	(0.020)	(0.019)	(0.020)	(0.019)	(0.020)	(0.019)	(0.020)	(0.019)	(0.020)
Age	−0.005	−0.006	−0.004	−0.005	−0.004	−0.005	−0.005	−0.006	−0.003	−0.005
	(0.005)	(0.005)	(0.005)	(0.005)	(0.005)	(0.005)	(0.005)	(0.005)	(0.005)	(0.005)
Age square	0.000	0.000+	0.000	0.000+	0.000+	0.000+	0.000	0.000+	0.000	0.000+
	(0.000)	(0.000)	(0.000)	(0.000)	(0.000)	(0.000)	(0.000)	(0.000)	(0.000)	(0.000)
Gender	−0.079 ***	−0.076 ***	−0.065 **	−0.063 **	−0.065 **	−0.063 **	−0.068 **	−0.066 **	−0.067 **	−0.065 **
	(0.022)	(0.022)	(0.022)	(0.022)	(0.022)	(0.022)	(0.022)	(0.022)	(0.022)	(0.022)
SES	−0.122 ***	−0.119 ***	−0.124 ***	−0.121 ***	−0.124 ***	−0.121 ***	−0.125 ***	−0.121 ***	−0.121 ***	−0.118 ***
	(0.012)	(0.012)	(0.012)	(0.012)	(0.012)	(0.012)	(0.012)	(0.012)	(0.012)	(0.012)
Satisfaction with social welfare services	0.063 ***	0.060 ***	0.064 ***	0.061 ***	0.064 ***	0.061 ***	0.064 ***	0.061 ***	0.063 ***	0.060 ***
	(0.005)	(0.006)	(0.005)	(0.006)	(0.005)	(0.006)	(0.005)	(0.006)	(0.005)	(0.006)
cons	2.879 ***	2.902 ***	2.880 ***	2.903 ***	2.886 ***	2.903 ***	2.944 ***	2.962 ***	2.838 ***	2.868 ***
	(0.134)	(0.132)	(0.141)	(0.139)	(0.139)	(0.137)	(0.131)	(0.129)	(0.136)	(0.134)
N	3162	3162	3162	3162	3162	3162	3162	3162	3162	3162
R2	0.133	0.135	0.132	0.134	0.132	0.134	0.132	0.134	0.134	0.136

Notes: For each of the variables, standard errors are in parentheses. * *p* < 0.05, ** *p* < 0.01, *** *p* < 0.001.

**Table 4:** Test of the mediating effect.

**Panel A: Testing the Mediating Effects of EQP 1**													
Variables		Outcome: EQP			Outcome: Happiness			Outcome: Happiness			Outcome: Happiness		
		(1)			(2)			(3)			(4)		
		beta	t	*p*	beta	t	*p*	beta	t	*p*	beta	t	*p*
Study		−0.016	−2.01	0.045				0.016	2.79	0.005	0.017	2.95	0.003
EQP1					0.052	3.6	0.000				0.053	3.69	0.000
Age		−0.012	−1.85	0.064	−0.004	−0.85	0.396	−0.003	−0.71	0.477	−0.003	−0.57	0.566
Age square		0.000	2.65	0.008	0.000	1.49	0.135	0.000	1.6	0.11	0.000	1.40	0.16
Gender		0.048	1.56	0.118	−0.066	−3.00	0.003	−0.064	−2.89	0.004	−0.066	−3.01	0.003
SES		0.011	0.64	0.522	−0.128	−9.85	0.000	−0.124	−9.4	0.000	−0.124	−9.5	0.000
Satisfaction with social welfare services		0.122	15.7	0	0.070	10.73	0.000	0.075	12.35	0.000	0.068	10.56	0.000
R2		0.098 ***			0.124 ***			0.121 ***			0.126 ***		
F		49.19			62.17			58.18			54.87		
**Panel B: Testing the Mediating Effects of EQP2**													
Variables		Outcome: EQP 2			Outcome: Happiness			Outcome: Happiness			Outcome: Happiness		
		(1)			(2)			(3)			(4)		
		beta	t	*p*	beta	t	*p*	beta	t	*p*	beta	t	*p*
Study		0.031	1.88	0.06				0.016	2.79	0.005	0.015	2.63	0.009
EQP 2					0.031	4.52	0.000				0.030	4.44	0.000
Age		0.047	3.29	0.001	−0.006	−1.25	0.21	−0.003	−0.71	0.477	−0.005	−1.01	0.314
Age square		0.000	−2.77	0.006	0.000	1.93	0.054	0.000	1.6	0.11	0.000	1.85	0.064
Gender		−0.019	−0.28	0.777	−0.063	−2.87	0.004	−0.064	−2.89	0.004	−0.063	−2.88	0.004
SES		−0.157	−4.00	0.000	−0.122	−9.37	0.000	−0.124	−9.4	0.000	−0.120	−9.07	0.000
Satisfaction with social welfare services		0.361	18.4	0.000	0.064	9.71	0.000	0.075	12.35	0.000	0.064	9.63	0.000
R2		0.174 ***			0.127 ***			0.121 ***			0.129 ***		
F		69.6			62.09			58.18			54.57		
**Panel C: Testing the Mediating Effects of EQP 1 and EQP 2 (bootstrap = 5000)**													
Mediator	Independent Variable	Conditional Indirect Effect			Boot SE			95% CI (Bias-Corrected and Accelerated)					
EQP 1	Study	−0.0009			0.00049			[−0.0020729, −0.0000925]					
EQP 2	Study	0.0009			0.0055			[0.0000344, 0.002255]					

Note: *** *p* < 0.001.

**Table 5:** Regression analysis results of the sense of environmental security moderate the mediation process.

	Step 1 (Outcome: EQP 1)			Step 2 (Outcome: Happiness)		
Predictor	beta	t	*p*	beta	t	*p*
Study	−0.100	−2.67	0.008	0.017	2.91	0.004
Sense of environmental security	0.330	7.62	0.000			
Study × Sense of environmental security	0.028	2.36	0.018			
EQP 1				0.053	4.16	0.000
Age	−0.013	−1.96	0.050	−0.003	−0.55	0.583
Age square	0.000	2.62	0.009	0.000	1.35	0.176
Gender	0.040	1.37	0.171	−0.066	−3.02	0.003
SES	0.023	1.42	0.157	−0.124	−10.04	0.000
Satisfaction with social welfare services	0.093	13.25	0.000	0.068	12.75	0.000
R2	0.1710			0.1261		
F	81.32 ***			65.04 ***		

Note: *** *p* < 0.001.

**Table 6:** The mediation effect of EQP 1 (bootstrap = 5000).

EQP1	Conditional Indirect Effect	SE	95% CI (Bias-Corrected and Accelerated)
M − SD	0.740	0.264	[0.213, 1.224]
M	0.867	0.307	[0.240, 1.403]
M + SD	0.994	0.351	[0.264, 1.584]

## Data Availability

The datasets used in the current study are available from the corresponding author upon reasonable request.
